# Lack of Galanin 3 Receptor Aggravates Murine Autoimmune Arthritis

**DOI:** 10.1007/s12031-016-0732-9

**Published:** 2016-03-03

**Authors:** Bálint Botz, Ágnes Kemény, Susanne M. Brunner, Felix Sternberg, Janka Csepregi, Attila Mócsai, Erika Pintér, Jason J. McDougall, Barbara Kofler, Zsuzsanna Helyes

**Affiliations:** 1 Molecular Pharmacology Research Team, Neuroscience Centre and János Szentágothai Research Centre, Department of Pharmacology and Pharmacotherapy, Medical School, University of Pécs, Pécs, Hungary; 2 MTA-PTE NAP B Chronic Pain Research Group, Pécs, Hungary; 3 Laura Bassi Centre of Expertise-THERAPEP, Research Program for Receptor Biochemistry and Tumor Metabolism, Department of Pediatrics, Paracelsus Medical University, Muellner Hauptstr. 48, 5020 Salzburg, Austria; 4 Department of Physiology, Semmelweis University School of Medicine and MTA-SE „Lendület” Inflammation Physiology Research Group, Budapest, Hungary; 5 Departments of Pharmacology and Anesthesia, Pain Management & Perioperative Medicine, Dalhousie University, Halifax, NS Canada

**Keywords:** Neuropeptide, Galanin, Inflammation, Arthritis, Plasma leakage, Myeloperoxidase

## Abstract

Neurogenic inflammation mediated by peptidergic sensory nerves has a crucial impact on the pathogenesis of various joint diseases. Galanin is a regulatory sensory neuropeptide, which has been shown to attenuate neurogenic inflammation, modulate neutrophil activation, and be involved in the development of adjuvant arthritis, but our current understanding about its targets and physiological importance is incomplete. Among the receptors of galanin (GAL_1_–_3_), GAL_3_ has been found to be the most abundantly expressed in the vasculature and on the surface of some immune cells. However, since there are minimal in vivo data on the role of GAL_3_ in joint diseases, we analyzed its involvement in different inflammatory mechanisms of the K/BxN serum transfer-model of autoimmune arthritis employing *GAL*_*3*_ gene-deficient mice. After arthritis induction, GAL_3_ knockouts demonstrated increased clinical disease severity and earlier hindlimb edema than wild types. Vascular hyperpermeability determined by in vivo fluorescence imaging was also elevated compared to the wild-type controls. However, neutrophil accumulation detected by in vivo luminescence imaging or arthritic mechanical hyperalgesia was not altered by the lack of the GAL_3_ receptor. Our findings suggest that GAL_3_ has anti-inflammatory properties in joints by inhibiting vascular hyperpermeability and consequent edema formation.

## Introduction

Rheumatoid arthritis (RA) is a chronic, inflammatory autoimmune disease that primarily affects the synovial joints manifesting as pain, stiffness, and synovitis. Edema formation and tenderness around the affected joints are characteristics of the early phase of the disease which is associated with progressive, irreversible degeneration and bone remodeling in later stages. Despite the increasing number of novel drugs introduced to treat RA (Smolen et al. 2007), long-term therapeutic relief is still poor for most patients (Jones et al. 2003). As such, there is still a great need for the identification of novel targets and the subsequent development of efficacious and safe drugs.

While immunological aspects of the pathological mechanisms of RA have been well described, components of the nervous system have long been believed to be potential contributors to immune-mediated disease conditions and recent evidence has corroborated this assertion (Levine et al. 1985; Bozic et al. 1996; Brogden et al. 2005; Kioussis and Pachnis 2009). The activation of the sensory nervous system triggers the peripheral release of several peptide and non-peptide mediators. These can have both anti- or proinflammatory effects, thereby regulating the inflammatory microenvironment by altering blood flow, vascular permeability, and leukocyte activity. This process, known as neurogenic inflammation (Holzer 1998), has been described clinically, where damage to the central or peripheral nervous system can dramatically alter the course of inflammatory diseases, such as RA (Thompson and Bywaters 1962; Kim et al. 2012). It has also been shown in preclinical models that an intact innervation is necessary for the development of joint inflammation, suggesting a pivotal role of pro-inflammatory neurotransmitters (McDougall et al. 1999; Kane et al. 2005; Stangenberg et al. 2014). In contrast, however, the defunctionalization of peptidergic sensory afferents can also lead to increased inflammation in several animal models (Helyes et al. 2004; Borbely et al. 2015). Thus, the role of neurogenically released peptide mediators in joints can be somewhat divergent with some neuropeptides being clearly pro-inflammatory (e.g., substance P) (McDougall et al. 1994), while others have anti-inflammatory properties (e.g., pituitary adenylate cyclase-activating polypeptide or endomorphin-1) (McDougall et al. 2004; Botz et al. 2014). The role of numerous other peptide mediators and their receptors including the galanin family in RA has yet to be examined.

Galanin is a sensory neuropeptide with a length of 30 (29 in rodents) amino acids that is ubiquitously expressed in both the central and peripheral nervous systems and has numerous biological and physiological functions. Three galanin receptors (GAL_1–3_) have been identified, which are all G-protein-coupled receptors (GPCR) showing distinct differences regarding their tissue expression pattern (Lang et al. 2015). Both GAL_1_ and GAL_2_ are present in abundance throughout the central nervous system, whereas GAL_3_ expression is much more restricted to the hippocampus (Mennicken et al. 2002). In non-neural tissues, GAL_2_ and GAL_3_ are predominantly expressed (Santic et al. 2007). The functional coupling and signal transduction pathways of the three galanin receptors are substantially different, giving rise to the great variety of galanin-mediated effects. While it was shown that GAL_1_ mainly signals via G_i/o_-type G proteins and GAL_2_-mediated effects involve multiple classes of G proteins, the signaling pathways of GAL_3_ are not well understood (Lang et al. 2015). Galanin has been shown to be upregulated following nerve damage (Ch’ng et al. 1985; Skofitsch and Jacobowitz 1985; Hokfelt et al. 1987) and has been implicated in nociception (Liu and Hokfelt 2002; Lang et al. 2015), as both galanin and its receptors are expressed in dorsal root ganglia and in the spinal dorsal horn (Landry et al. 2005). Galanin knockout mice exhibit increased sensitivity to mechanical and thermal stimuli, whereas galanin-overexpressing mice show an increased thermonociceptive threshold (Blakeman et al. 2001; Holmes et al. 2003). Furthermore, the galanin peptide family is undoubtedly involved in the regulation of inflammatory processes with the galanin system being upregulated in the central and peripheral nervous systems in response to inflammation (Ji et al. 1995). Galanin has been shown to have anti-inflammatory and most importantly anti-edema effects in animal models of inflammation (Lang and Kofler 2011). GAL_3_ was found to be active in the dermal microvasculature, as treatment with its selective small molecule antagonist SNAP 37889 dose-dependently blocked the anti-edema effect of galanin (Schmidhuber et al. 2009). Furthermore, galanin gene-deficient mice lack neurogenic inflammatory responses and have impaired neutrophil recruitment into inflamed tissues (Schmidhuber et al. 2008). According to recent data, GAL_2_ but not GAL_1_ is expressed on both human and murine neutrophils, whereas galanin and GAL_3_ are expressed on murine neutrophils only. Additionally, it has been reported that galanin can act as a modulator of cytokine-induced neutrophil activation (Locker et al. 2015). Galanin itself has been implicated in arthritis as an endogenous regulatory mediator. Several studies reported a change in galanin mRNA levels, galanin-like immunoreactivity, and galanin peptide levels, respectively, in the rat dorsal horn of the spinal cord, dorsal root ganglia, and joint tissue after experimentally induced adjuvant arthritis (Hope et al. 1994; Calza et al. 1998; Calza et al. 2000; Qinyang et al. 2004). Hence, the galanin system poses a novel target for alternative treatment stategies for RA; however, no studies have been conducted identifying the relevant galanin receptor subtype.

The K/BxN serum-transfer model of autoimmune arthritis (Kouskoff et al. 1996; Korganow et al. 1999) mimics numerous aspects of RA in humans. The model produces a transient, but profuse polyarthritis following systemic administration of exogenous antibodies (anti-glucose phosphate isomerase) with the involvement of neutrophils (but not of T/B cells). This arthritis model also has a distinct neurogenic component (Korganow et al. 1999; Botz et al. 2014; Stangenberg et al. 2014; Borbely et al. 2015).

Since galanin participates in the pathogenesis of arthritis and because GAL_3_ is expressed on murine neutrophils and has been shown to influence vascular components of inflammatory processes, we hypothesize that GAL_3_ is involved in inflammatory joint diseases. Therefore, the aim of the present study was to elucidate if GAL_3_ plays a role in the K/BxN serum transfer model of autoimmune arthritis.

## Materials and Methods

### Experimental Animals

Experiments were conducted using 12–14-week-old male *GAL*_*3*_ gene-deficient (GAL_3_^−/−^) mice and age-matched wild-type (GAL_3_^+/+^) controls (body weight 25–30 g). GAL_3_^−/−^ (LEXKO-230) mice were obtained from the European Mouse Mutant Archive. The mice were generated by homologous recombination with targeting both coding exons. The mouse line was backcrossed onto a C57BL/6 lineage for at least seven additional generations and was maintained on this background. The successful knockout of the *GAL*_*3*_ gene has been established recently (Brunner et al. 2014). All animals were GAL_3_ genotyped before the experiments. Animals were bred and kept in the Laboratory Animal House of the Department of Pharmacology and Pharmacotherapy of the University of Pécs, at 24–25 °C ambient temperature, and provided with standard rodent chow and water ad libitum under 12-h light-dark cycles.

### The K/BxN Serum-Transfer Induced Inflammatory Arthritis

K/BxN mice express a transgenic T cell receptor and the MHC class II allele A^g7^. This leads to the production of autoantibodies against the enzyme glucose-6-phosphate isomerase and consequent development of progressive polyarthritis. Transfer of K/BxN serum into mice elicits a robust, albeit transient, polyarthritis. This serum transfer model mimics predominantly the effector phase of RA and depends mainly on mast cells and neutrophils, but not on lymphocytes (Monach et al. 2008). The sera of transgene-positive (K/BxN) and negative (BxN) mice were harvested, pooled, and stored at −80 °C as described earlier (Korganow et al. 1999; Jakus et al. 2009). Arthritis was induced by a single intraperitoneal (i.p.) injection of 300 μl of the arthritogenic (K/BxN) or control (BxN) serum.

### Evaluation of Disease Severity and Hindpaw Edema

Arthritis severity was evaluated daily until 13 days post serum injection by semiquantitative scoring between 0 and 10 based on two key signs of inflammation: edema and hyperemia. A score of ≤0.5 represented a normal hindlimb, and 10 refers to the most severe level of joint inflammation with accompanying gait abnormality. Hindlimb edema was also monitored repeatedly (on days 0, 2, 4, 6, 8, and 11 post serum injection) by plethysmometry (Ugo Basile, Comerio, Italy).

### Assessment of Mechanonociception and Joint Function

The mechanical hyperalgesia of the hindpaw was measured every second day by dynamic plantar esthesiometry (Ugo Basile). Mechanonociceptive threshold was expressed as percentage of pretreatment controls. Grasping ability was tested repeatedly (on days 0, 2, 4, and 6 post serum injection) by placing the mice on a horizontal wire-grid, which was then turned over and maintained in this position for 30 s or until the animal fell (Jakus et al. 2009).

### *In vivo* Fluorescence Imaging of Plasma Leakage

On days 0, 1, and 5, post serum injection mice received a retroorbital injection of the fluorescence contrast agent indocyanine-green (0.5 mg/kg body weight) dissolved in 5 % *w*/*v* Kolliphor HS 15 solution (Sigma-Aldrich) (Kirchherr et al. 2009) under ketamine-xylazine anesthesia (ketamine 100 mg/kg; xylazine 5 mg/kg body weight i.p.). It has been demonstrated that this micellar fluorescent contrast agent enables sensitive noninvasive detection of microvascular extravasation (Botz et al. 2014). The underlying principle of this technique is that the labeled micelles serve as nanoprobes that can leave the microvasculature of inflamed tissues, but not the intact vessels due to their size (∼10 nm). This results in retention and buildup of fluorophore in the inflamed tissue, culminating in increased fluorescence that correlates with the severity of the disease (Kenne and Lindbom 2011). Animals were imaged 20 min post-injection using the IVIS Lumina II system (Perkin-Elmer, Waltham, MA, USA). Imaging parameters were set to the following: auto acquisition time, F/Stop = 1, Binning = 2. The excitation and emission filters were 745/800 nm. Data were analyzed using the Living Image® software; regions of interests (ROIs) were drawn around the hind limbs. A calibrated unit of fluorescence, the radiant efficiency ([photons/s/cm^2^/sr]/[μW/cm^2^]) originating from the ROIs was used for further analysis.

### *In vivo* Bioluminescence Imaging of Neutrophil Myeloperoxidase Activity

Luminol (5-amino-2,3-dihydro-1,4-phthalazine-dione) is a chemiluminescent reactive oxygen species sensor, which in vivo requires both the superoxide-production of nicotinamide adenine dinucleotide phosphate oxidase and the activity of the myeloperoxidase (MPO) enzyme. The MPO-dependent nature of luminol makes it a suitable chemiluminescent tracer to image the activity of this enzyme, and thereby the functioning of neutrophils *in vivo*, as most of the MPO-activity is localized in the phagosomes of those cells during inflammation (Gross et al. 2009; Tseng and Kung 2012). On days 0, 1, and 5, post serum injection mice received an i.p. injection of 20 mg/ml PBS-based solution of sodium-luminol (Sigma-Aldrich) at a dose of 150 mg/kg, and were imaged 10 min post-injection using the IVIS Lumina II. Acquisition time was 60 s, F/stop = 1, Binning = 8. ROIs were applied as previously described and luminescence was expressed as total radiance (total photon flux/s).

### Histology

Joint samples were harvested on day 14 following euthanasia by sodium pentobarbital (100 mg/kg i.p.). Ankle joints were fixed in 40 mg/ml buffered formaldehyde, dehydrated using ethanol and xylol, and finally decalcified with EDTA. The samples were embedded in paraffin, sectioned (3–5 μm), and stained with fast green and safranine O.

### Dynamic Mass Redistribution Assay

Murine polymorphonuclear neutrophils were isolated from the bone marrow as described previously (Locker et al. 2015). Cells were resuspended in Hank’s Balanced Salt Solution containing magnesium and calcium [HBSS (+/+)] (Gibco), diluted in HBSS (+/+) containing 20 mM HEPES (Gibco), and then seeded onto EnSpire LFC-384 well plates coated with fibronectin (Perkin-Elmer) at a density to achieve a confluent monolayer (60,000–80,000 cells/well). The plates were centrifuged for 10 s at 1000*g* and equilibrated for 1 h in the EnSpire machine (Perkin-Elmer). First, the pretreatment baseline was acquired by measuring 4 repeats (30 s each) followed by the addition of compounds [KC (the murine homolog of IL-8) (1 pM–100 nM), KC (1 pM–100 nM) with 10 μM galanin, KC (1 pM–100 nM) with 1 μM galanin, and KC (1 pM–100 nM) with 0.1 μM galanin]. The plate was measured for 20 repeats. Each experiment was carried out in triplicate, and the mean was used to generate the dynamic mass redistribution (DMR) traces. The half maximal effective concentration (EC_50_) of KC was then calculated.

### Expression Analysis

Expression profiles of galanin and its receptors was performed in 16–24-week-old male GAL_3_^−/−^ and GAL_3_^+/+^ mice as described previously (Brunner et al. 2014). Briefly, mice were euthanized by CO_2_ overdose and cervical dislocation. Tissue was dissected and immediately snap-frozen in liquid nitrogen. RNA isolation was performed with TRI Reagent (Molecular Research Center, Inc.) according to the manufacturer’s instructions. Synthesis of cDNA was performed by use of random hexamer primers and Maxima reverse transcriptase (Thermo Scientific) according to the manufacturer’s instructions. Expression profiles of galanin and GAL_1–3_ were quantified by quantitative real-time PCR using B-R SYBR Green SuperMix for iQ (Quanta BioSciences, Inc.) and iCycler iQ real-time PCR detection system (Bio-Rad Laboratories). Primer sequences and cycling conditions are taken from Brunner et al. (2014).

### Statistical Analysis

Results are expressed as mean ± SEM. Statistical evaluation was performed by Graphpad Prism®. Functional data were analyzed by two-way ANOVA + Tukey’s multiple comparison test, grasping ability results by logrank test, imaging, DMR assay, and expression analysis results by Student’s unpaired *t* test. *p* values below 0.05 were considered significant.

## Results

### More Severe Arthritis Progression and Accelerated Edema Formation in GAL_3_^−/−^ Mice

**Fig. 1:**
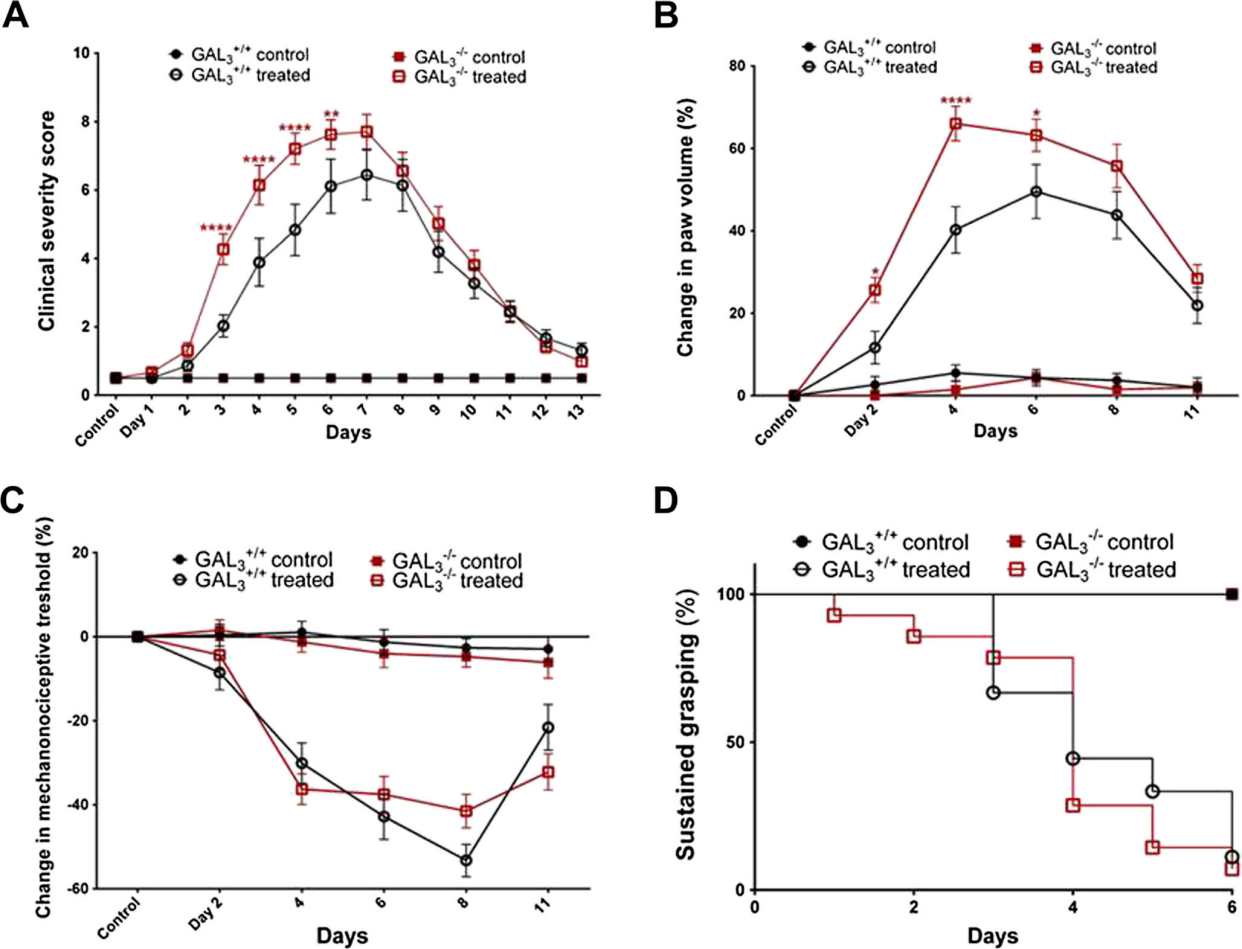
GAL_3_-deficiency leads to increased edema and inflammation without affecting nociception or motor functions. **a** Change of disease severity in wild-type (GAL_3_^+/+^) and gene-deficient (GAL_3_^−/−^) mice. **b** Hindlimb edema measured by plethysmometry. **c** Arthritic mechanical hyperalgesia measured by plantar esthesiometry. **d** Motor impairment measured by wire grid grip test and plotted as a survival curve. Two-way ANOVA + Tukey’s multiple comparison test, survival curve: logrank test. Controls: *n* = 6–7, arthritic groups: *n* = 9–12. *p < 0.05, **p < 0.01, ****p < 0.0001 vs. respective wild type

Joint inflammation occurred in GAL_3_^+/+^ and GAL_3_^−/−^ mice with similar kinetics and peaks at day 7, but GAL_3_^−/−^ mice showed a more severe arthritis phenotype compared to wild types (peak difference observed from day 3 to 5 with *p* < 0.0001) (Fig. 1a). The plethysmometric determination of the hindpaw volume revealed an earlier peak of edema formation in GAL_3_^−/−^ mice (GAL_3_^−/−^ day 4 at 65 %; GAL_3_^+/+^ day 6 at 50 %), and a more robust plasma extravasation on days 2 to 6 in GAL_3_^−/−^ mice compared to GAL_3_^+/+^ wild types (peak differences on day 4 *p* < 0.0001 and day 6 *p* = 0.0238) (Fig. 1b).

### GAL_3_ Deficiency Does not Influence Nociception or Motor Performance in the Arthritis Model

As RA is known to affect nociception and motor performance, we tested whether GAL_3_ is involved in pain perception. A considerable and similar mechanonociceptive threshold drop of about 40–50 % was observed in both the GAL_3_^−/−^ and GAL_3_^+/+^ mice on day 8 of the experiment (Fig. 1c), indicating increased pain in both groups. Grasping ability also decreased steadily following serum injection. This reached a peak by day 6 as almost all K/BxN serum-treated GAL_3_^−/−^ and GAL_3_^+/+^ animals became unable to maintain their position on the grid for the duration of the test period (Fig. 1d).

### Increased Early-Phase Arthritic Vascular Hyperpermeability in GAL_3_ Knockouts

**Fig. 2:**
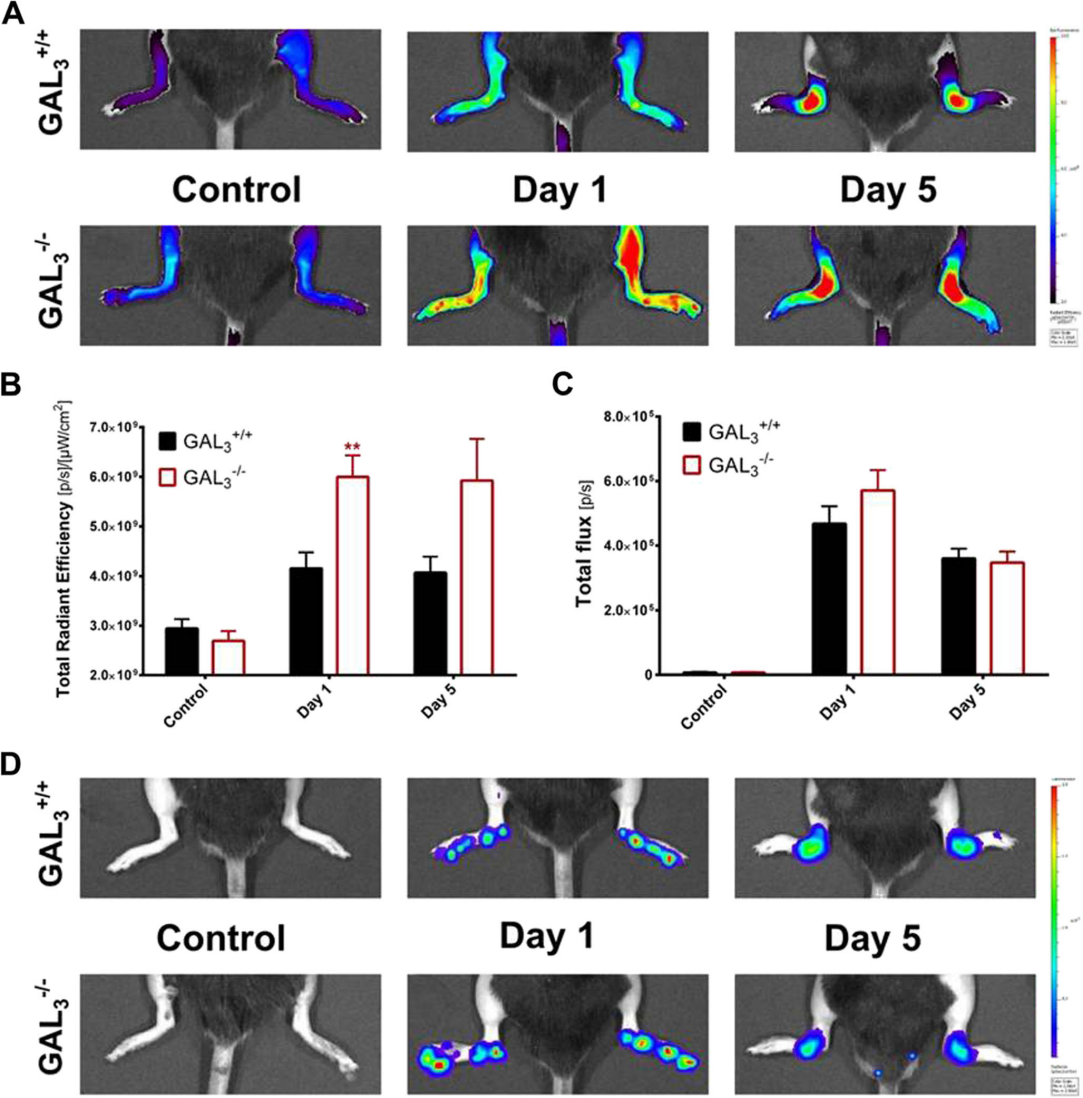
GAL_3_ deficiency results in increased and early vascular hyperpermebility in arthritis, without directly affecting neutrophil ROS production. **a** Representative in vivo fluorescence images highlighting indocyanine-green extravasation. **b** Quantification of normalized fluorescence in the hind paws representing the degree of vascular leakiness (*n* = 5–6). **c** Quantification of normalized luminescence in the hind limbs showing MPO-derived ROS-production of neutrophils (controls: *n* = 6–7, arthritic groups: *n* = 9–12). **d** Representative *in vivo* luminescence images. Student’s unpaired *t* test, **p < 0.01 vs. respective wild type

Plasma leakage after arthritis induction was assessed by in vivo fluorescence imaging. Pretreatment control fluorescence was comparable in GAL_3_^−/−^ and GAL_3_^+/+^ mice. The degree of plasma extravasation increased sharply upon K/BxN serum transfer in both groups, peaking 24 h after arthritis induction in the hyperacute phase of the disease. GAL_3_^−/−^ mice exhibited 40 % greater vascular hyperpermeability (*p* < 0.01) compared to wild types. This significant difference in plasma leakage resolved by day 5, albeit the overall degree of vessel hyperpermeability remained similar in the two groups (Fig. 2a, b).

### Similar MPO-Activity and Joint Damage in Arthritic GAL_3_^−/−^ Mice

**Fig. 3:**
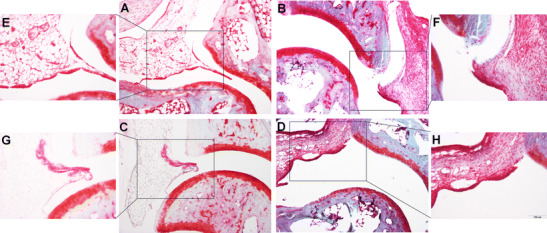
Representative microphotographs of the joint samples of GAL_3_^+/+^ (**a**, **b**, **e**, **f**) and GAL_3_^−/−^ (**c**, **d**, **g**, **h**) mice taken on day 14 after arthritis induction. The adipocyte-rich periarticular connective tissue of the control groups (**a**, **c**, **e**, **g**) (illustrated by the frame) was replaced with a dense fibroblastic scar tissue, with a limited presence of inflammatory cells in the arthritic groups (**b**, **d**, **f**, **h**). No difference was observed in these respects between the study groups [fast green and safranin O staining, ×100 (**a**–**d**) and ×200 (**e**–**h**) magnification]

As neutrophil recruitment is a characteristic of the K/BxN model of autoimmune arthritis, we aimed to evaluate the involvement of GAL_3_ in activating neutrophils using in vivo bioluminescence imaging. Baseline luminol bioluminescence was negligible in both groups. Following K/BxN serum-transfer, the MPO-derived ROS production increased dramatically in both GAL_3_^−/−^ and GAL_3_^+/+^ animals, reaching a peak on day 1. Neutrophil ROS production decreased considerably by day 5, indicating that the disease was already in transition from a neutrophil-dominated acute phase into the chronic macrophage-mediated stage. However, MPO-activity did not differ significantly between knockouts and wild types on day 1 or 5 (Fig. 2c, d). Histological samples were harvested 14 days after serum transfer revealed a similar phenomenon. The synovial lining was thickened, and the normally adipocyte-rich periarticular connective tissue was replaced with a dense fibroblastic scar tissue, with limited inflammatory cell infiltration. No remarkable difference was observed in these respects between the study groups, in agreement with the absent functional difference at this stage of the disease (Fig. 3).

### Sensibilization of Neutrophils by Galanin Is GAL_3_ Independent

**Fig. 4:**
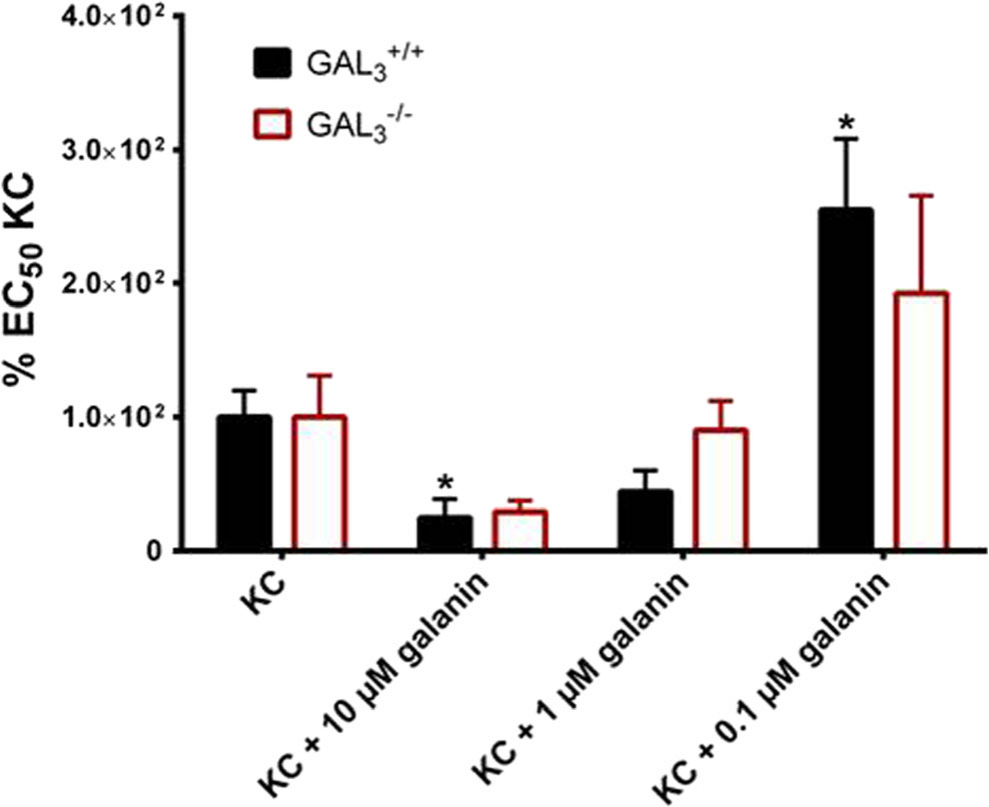
Comparison of the relative EC_50_ of BM-PMNs from wild-type C57BL/6 mice (Locker et al. 2015) and BM-PMNs from *GAL*_*3*_ gene-deficient animals. The cells were treated with KC, the murine homolog of IL-8, in the presence of a fixed concentration of galanin. Data were normalized to KC treatment alone, which was set to 100 %. Data were analyzed with Student’s *t* test for unpaired and paired comparisons (with Welch correction), respectively. *p < 0.05 vs. respective KC alone (*n* = 8–9)

Since no difference in neutrophil infiltration could be observed between wild-type and knockout animals, we tested if the recently reported modulation of neutrophil activation by galanin is GAL_3_ dependent (Locker et al. 2015). We found that in polymorphonuclear neutrophils isolated from the bone marrow (BM-PMNs) of GAL_3_^−/−^ mice, galanin co-treatment resulted in a similar dose-dependent shift of the DMR and consequently a similar modulation of the EC_50_ of KC, the murine homolog of IL-8, compared to wild-type mice (Fig. 4). This finding is in agreement with the in vivo data presented here, showing that GAL_3_ is not affecting neutrophil function.

### Expression Profiles of the Galanin System Are Not Affected by the GAL_3_ Knockout

Since compensatory upregulation of galanin and the other galanin receptors in the GAL_3_^−/−^ mice could potentially influence our findings, we analyzed expression levels of galanin and its receptors in the spleen, lung, kidney, liver, and testes of GAL_3_^−/−^ and GAL_3_^+/+^ mice. We found that deltaCt values of galanin system genes compared to the housekeeping gene HPRT were similar in GAL_3_^−/−^ and GAL_3_^+/+^ animals, indicating no compensatory mechanisms in peripheral tissues of GAL_3_-deficient mice (Table 1).

**Table 1:** Expression levels of galanin system genes displayed as deltaCt values compared to the housekeeping gene HPRT in GAL_3_^+/+^ and GAL_3_^−/−^ mice. Data are represented as mean ± SEM, *n* = 2–4

	Gene	GAL		GAL_1_		GAL_2_		GAL_3_	
Tissue	Genotype	∆Ct	*p* value	∆Ct	*p* value	∆Ct	*p* value	∆Ct	*p* value
Spleen	GAL_3_^+/+^	4.0 ± 1.5	0.347	10.0 ± 2.1	0.459	14.8 ± 0.5	0.058	12.0 ± 0.5	n.a.
	GAL_3_^−/−^	5.8 ± 0.9		12.0 ± 1.4		16.5 ± 0.5		n.d.	
Lung	GAL_3_^+/+^	5.4 ± 0.2	0.180	11.3 ± 0.8	0.999	12.8 ± 0.1	0.644	10.3 ± 0.5	n.a.
	GAL_3_^−/−^	5.9 ± 0.3		11.3 ± 1.0		12.3 ± 0.5		n.d.	
Kidney	GAL_3_^+/+^	13.2 ± 0.4	0.412	14.4 ± 1.0	0.723	11.1 ± 0.3	0.568	9.4 ± 0.7	n.a.
	GAL_3_^−/−^	12.6 ± 0.6		13.9 ± 0.4		10.7 ± 0.5		n.d.	
Testes	GAL_3_^+/+^	5.5 ± 0.5	0.486	n.d.	n.a.	7.5 ± 0.4	0.609	13.4 ± 0.2	n.a.
	GAL_3_^−/−^	5.9 ± 0.3		n.d.		7.7 ± 0.1		n.d.	
Liver	GAL_3_^+/+^	16.7 ± 0.9	0.860	n.d.	n.a.	15.2 ± 0.6	0.357	15.6 ± 0.7	n.a.
	GAL_3_^−/−^	17.0 ± 1.3		n.d.		16.5 ± 1.5		n.d.	

*n.d.* not detectable, *n.a.* not applicable

## Discussion

Our results suggest a modest involvement of GAL_3_ receptor signaling in neurogenic inflammatory arthritis by decreasing microvascular leakage and consequent edema formation.

The vasoregulatory role of peripherally released galanin has been investigated previously where it was found to be able to inhibit histamine-induced edema formation in the skin (Jancso et al. 2000). Later results also revealed that GAL_2_ and GAL_3,_ but not GAL_1,_ are expressed in the skin and the anti-edema effect of galanin is presumably mediated through these receptors present on perivascular neural, but not endothelial or smooth muscle tissues (Schmidhuber et al. 2007). Consistent with our finding in K/BxN-induced arthritis, it was reported that inhibition of GAL_3_ signaling with SNAP 37889 also resulted in elevated edema formation (Schmidhuber et al. 2009). Our results suggest that GAL_3_-agonism is an endogenous protective mechanism in immune-mediated arthritis driven by neurogenic factors. Previously, it was demonstrated that galanin immunoreactivity increases in the dorsal root ganglion during experimental arthritis (Calza et al. 2000). In adjuvant arthritis of the rat, others found a decrease in galanin immunoreactivity in the sciatic nerve and macrophage-like cells, whereas it was found to be elevated, e.g., in fibroblasts, osteoblasts, and the polymorphonuclear lineage cells of the bone marrow (Qinyang et al. 2004). However, the observed difference in our model is not necessarily galanin-mediated, as galanin-like peptide (GALP) is also able to activate the GAL_3_ receptor (Lang et al. 2015). Furthermore, it has been shown recently that the novel neuropeptide spexin is a more potent agonist of GAL_3_ than galanin itself (Kim et al. 2014). Unfortunately, it has not been shown so far if spexin is able to activate GAL_3_ in vivo or if it is expressed in murine joints or neutrophils. Therefore, it is not possible to state which of these three peptides is responsible for the observed GAL_3_-mediated effects in K/BxN induced arthritis.

We did not observe any difference in neutrophil MPO-activity, suggesting that GAL_3_-deficiency in vivo may not influence the function of these immune cells. Indeed, recent results show that GAL_3_ is not expressed in mature human blood neutrophils unlike murine bone marrow neutrophils. Whether this discrepancy reflects a species-difference or the difference between the sites of collection (peripheral blood vs. bone marrow) remains to be addressed. Galanin was also found to modulate the sensitivity of neutrophils isolated from the murine bone marrow towards KC, the murine homolog of IL-8 (Locker et al. 2015). We show here that this modulation is independent of the GAL_3_ genotype, supporting the finding that neutrophil activation is not dependent on GAL_3_ expression, at least not in K/BxN-induced murine arthritis. Interestingly, another study found that in a mouse model of acute pancreatitis, GAL_3_ antagonism by the selective nonpeptide antagonist SNAP 37889 ameliorated disease severity (Barreto et al. 2011). However, more recently, SNAP 37889 has been found to be cytotoxic in a variety of cell types, including, but not limited to, myeloid lineages. Since this effect is GAL_3_-independent (Koller et al. 2015), results obtained with SNAP 37889 have to be interpreted with care.

The lack of effect of *GAL*_*3*_ gene-deletion on mechanical hyperalgesia and accompanying loss of grasping function is supported by earlier findings implicating GAL_1_ and GAL_2_ but not GAL_3_ in nociceptive transmission. Since GAL_3_ shows only a very limited expression in the nervous system, this observation is in agreement with earlier results (Landry et al. 2005; Lang et al. 2015).

In this study, we also found no evidence that compensatory mechanisms of the galanin system occur in peripheral tissues of GAL_3_^−/−^ animals. Previously, Brunner and coworkers also reported no change in expression levels of the galanin system in different brain regions of these mice (Brunner et al. 2014). Therefore, compensatory mechanisms of the galanin system in GAL_3_^−/−^ mice can be excluded. However, we did not elucidate whether expression levels of galanin signaling elements are altered in the present mouse model and which signaling pathways are involved in the observed GAL_3_-mediated effects. Besides, signaling properties of GAL_3_ are still poorly defined. One explanation for this gap in knowledge is the lack of cell lines which endogenously express GAL_3_ only. Additionally, overexpression of GAL_3_ in different cell lines leads to the translation of the protein mainly as intracellular high molecular weight protein aggregates while omitting functional activation by exogenous galanin (Robinson et al. 2013; Lang et al. 2015). *In vivo*, GAL_3_ might interact with other GPCRs or arrestins etc. which stabilize GAL_3_ on the membrane. However, to our knowledge, there are no data available supporting this theory.

In conclusion, our findings suggest that GAL_3_ is a potential target through which galanin can reduce joint swelling, but not nociception. Since the K/BxN serum-transfer model depends on an intact innervation of the hindlimb and involves early neurogenic vasodilation (Binstadt et al. 2006; Stangenberg et al. 2014), activation of the GAL_3_ receptor may offset and limit the extent of neurogenic inflammation in joints. However, GAL_3_ is not a *sine qua non* of the inflammatory cascade due to the functional redundancy of sensory neuropeptides on a functional level, and also because GAL_3_-activation is responsible for only a fraction of the beneficial effects of galaninergic mediators. The anti-inflammatory effect of galanin peptides is mediated via multiple receptors, and GAL_3_-activation does play a role in the attenuation of the vascular component of nerve-driven inflammation. Thus, affinity towards GAL_3_ would be a desirable attribute for the development of effective anti-edema galanin-analogs.
